# Impaired polymorphonuclear neutrophils in the oral cavity of edentulous individuals

**DOI:** 10.1111/eos.12367

**Published:** 2017-08-19

**Authors:** Patrick Rijkschroeff, Bruno G. Loos, Elena A. Nicu

**Affiliations:** ^1^ Department of Periodontology Academic Centre for Dentistry Amsterdam Amsterdam the Netherlands

**Keywords:** apoptosis, granulocytes, innate immunity, oral health, reactive oxygen species

## Abstract

Oral health is characterized by functional oral polymorphonuclear neutrophils (oPMNs). Edentulism might be associated with a loss of oPMNs because these cells enter the oral cavity primarily through the gingival crevices. The main aim of this study was to investigate the numbers of oPMNs in rinse samples obtained from edentulous (*n* = 21) and dentate (*n* = 20) subjects. A second study aim was to investigate possible differences between oPMNs and peripheral blood polymorphonuclear neutrophils (cPMNs). Apoptosis/necrosis and cell‐activation markers (CD11b, CD63 and CD66b) were analyzed using flow cytometry. Reactive oxygen species (ROS) production was determined either without stimulation (constitutive) or in response to 10 *μ*M phorbol myristate acetate or *Fusobacterium nucleatum*. The edentulous subjects presented with lower oPMN counts and higher percentages of apoptotic/necrotic oPMNs compared with dentate subjects. Furthermore, oPMNs from edentulous donors expressed low levels of all three activation markers and low constitutive ROS. In contrast, oPMNs from dentate subjects expressed high levels of all three activation markers and a higher level of constitutive ROS than cPMNs. When challenged, oPMNs from edentulous subjects showed no upregulation in ROS production, whereas oPMNs from dentate subjects retained their ability to respond to stimulation. The functional characteristics of cPMNs were comparable between edentulous and dentate subjects. This study demonstrates that despite having functional cPMNs, edentulous subjects have low oPMN numbers that are functionally impaired.

Over the past century, the number of elderly people has significantly increased 1. Edentulism is prevalent among elderly people worldwide and is highly associated with socio‐economic status 1, 2, 3. The World Health Organization reported that within the European region, over 30% of people 65–74 yr of age have lost all their natural teeth 4. Denture use is common among the elderly in the Netherlands. In 2009, approximately 41% of the Dutch population over 65 yr of age wore full dentures and 34% wore partial dentures, according to Statistics Netherlands (CBS) 5. Several studies reported that the patients’ quality of life decreases with tooth loss. Edentulous subjects wearing full dentures experience more psychological discomfort and social disability compared with patients with partial dentures or with dentate subjects, in addition to oral discomfort, such as functional limitations and physical pain 6, 7, 8.

Currently, oral health is becoming increasingly recognized as imperative for maintaining systemic health 9, 10. Innate immune responses play a crucial role in the equilibrium within the oral cavity 11, 12. Important innate immune cells are the polymorphonuclear neutrophils (PMNs). They are the predominant leukocyte effector cells in the blood circulation, which can migrate to tissues in response to chemoattractant molecules such as lipopolysaccharide or lipoteichoic acid (released by microorganisms) or to products released from the host cells following tissue damage 13.

In a recent study, oral PMNs (oPMNs) were investigated in a group of orally and systemically healthy dentate individuals 14. Viable PMNs were found to be capable of performing innate immune functions, such as active degranulation and production of reactive oxygen species (ROS). A few early studies investigated leukocyte counts in the oral cavity of edentulous subjects; however, limited knowledge exists concerning PMN functionality in the edentulous oral cavity 15, 16, 17.

The first aim of the current study was to investigate the presence of oPMNs in edentulous subjects and to determine their functionality. To this end, we analyzed the numbers, membrane integrity, cell‐activation status, and reactive oxygen species (ROS) production capabilities of the oPMNs from a group of edentulous subjects and a comparable group of dentate subjects. The second aim was to investigate possible differences between the oPMNs and the PMNs from peripheral blood [i.e. the circulatory system PMNs (cPMNs)] in both groups.

## Material and methods

### Study population

For this case–control study, volunteers were recruited from the Academic Centre for Dentistry Amsterdam (ACTA), the Netherlands. Participants were either patients at the dental clinics or non‐professional personnel. All subjects presented without any history of pathological conditions related to altered PMN levels or function (such as hematological disorders, diabetes mellitus, antibiotic use within the last 6 months, recent history of illness or fever, allergies, alcoholism, and pregnancy). Furthermore, individuals with removable partial dentures, night guards, orthodontic banding, (peri)oral piercings, or apparent oral lesions were also excluded. The study was conducted in accordance with the Declaration of Helsinki (2008) of the World Medical Association and approximating Good Clinical Practice guidelines. Subjects were informed about the purpose of this study, received written information, and had given written consent before the start of the study. This study was approved by the Medical Ethics Committee of the VU University Medical Center (reference 2012‐210#B2012406) and was registered at the ISRCTN registry (ISRCTN 13234871).

Edentulous subjects could be included if they had been wearing a full denture for a minimum of 1 yr. Dentate subjects were screened for their periodontal condition using the Dutch Periodontal Screening Index 18, 19. The latter subjects were included if they had a minimum of 24 teeth and only if they showed a maximum probing depth of 4–5 mm in the absence of gingival recession. Additionally, bitewing radiographs were taken in order to confirm the absence of alveolar bone loss.

### Clinical collection procedure for oPMNs and cPMNs

The oPMNs and cPMNs were isolated and analyzed on the same day. The collection procedure was based on a previously described protocol 14. Briefly, in order to avoid contamination, oral rinse samples were taken before any other intra‐oral procedures. Edentulous subjects were asked to remove their dentures before supervised sample collection. All subjects were instructed not to gargle or to clear their throat during the sampling procedure. The oPMNs were obtained by four serial rinses of the oral cavity with 10 ml of sterile sodium chloride solution (0.9% NaCl) for 30 s, with an intermission of 4 min 30 s. The pooled sample was kept on ice in a 50‐ml centrifuge tube (Sigma‐Aldrich Chemie, Zwijndrecht, the Netherlands) until the end of the collection procedure. Venous blood samples were drawn from the antecubital fossa of all subjects into 9‐ml sodium heparin blood‐collection tubes (BD Vacutainer, Breda, the Netherlands) and were maintained at room temperature until processed using the PMN isolation procedure. The subsequent isolation procedures of the oPMNs and the cPMNs were carried out as previously described 14.

### Cell counts

The oPMN and the cPMN counts were made using a Muse Cell Analyzer (Merck Millipore, Darmstadt, Germany) with the Muse Count & Viability Kit (Merck Millipore), according to the manufacturer's instructions.

### Membrane integrity assessment

Morphological changes associated with apoptosis are a late manifestation of the programmed cell death pathway. During cell death, PMNs lose their membrane integrity. Propidium iodide (PI) is a fluorescent dye that can diffuse into cells and bind to DNA when membrane integrity is affected. Propidium iodide‐positive (PI^+^) cells are regarded as late apoptotic or necrotic 20, 21. In brief, oPMNs and cPMNs were incubated for 15 min in the dark at room temperature according to the manufacturer's instructions (BD Pharmingen FITC Annexin V Apoptosis Detection Kit; BD Biosciences, San Diego, CA, USA). Flow cytometric analysis was performed within 1 h using a BD Accuri C6 flow cytometer (BD Biosciences). The percentages of PI^+^ PMNs were calculated.

### Cell activation status

Low‐affinity immunoglobulin‐Fc*γ* receptor IIIb (CD16b) can be found on the surface membrane of mature PMNs and can be used as a PMN identification marker. Expression of receptors on the surface of PMNs was measured by flow cytometry. The PMNs were gated according to CD16 expression and the sideward scatter profile. To study PMN activation, samples were measured for the expression of phycoerythrin (PE)‐conjugated monoclonal antibodies (mAbs) (at a final concentration of 10 *μ*g ml^−1^) against CD11b and CD63 and for expression of fluorescein isothiocyanate (FITC)‐conjugated mAb against CD66b (all from BD Biosciences). Samples were incubated for 30 min on ice in the dark. Expression of cell‐surface markers was measured on the FL‐1 (Green) and FL‐2 (Red) channels and calculated as mean fluorescence intensity (MFI). Results were corrected for non‐specific binding, by subtracting the MFI values of the corresponding IgG1 isotype‐control antibodies (BD Biosciences).

### Bacterial culture

*Fusobacterium nucleatum* strain ATCC10953 was obtained commercially (DSMZ, Braunschweig, Germany) and grown anaerobically (80% nitrogen, 10% carbon dioxide, and 10% hydrogen) in a brain–heart infusion broth supplemented with 5 *μ*g ml^−1^ of hemin (Sigma‐Aldrich) and 1 *μ*g ml^−1^ of menadione (Sigma‐Aldrich). The bacteria were isolated from the broth cultures by centrifugation, washed twice in sterile PBS, then diluted with sterile PBS, to yield a final suspension of 4 × 10^8^ cells ml^−1^, which was stored at −20°C.

### ROS production assay

Dihydrorhodamine 123 (DHR) (Sigma‐Aldrich) is a non‐fluorescent probe that can diffuse passively across cell membranes; DHR can be oxidized by hydrogen peroxide and superoxide, and broken down into the green fluorescent rhodamine 123, which is detectable in the FL‐1 (Green) channel of a flow cytometer 22, 23. Unstimulated samples were incubated for 30 min with 2 mM DHR, in a shaking (50 r.p.m.) water bath at 37°C. Additionally, PMNs were stimulated with 10 *μ*M phorbol myristate acetate (PMA; Sigma‐Aldrich) or non‐opsonized *F. nucleatum* at a ratio of PMN:*Fn* of 1:20. Results were expressed as the MFI.

### Statistical analysis

Data analyses were performed using IBM SPSS 21.0 software (IBM, Chicago, IL, USA). Boxplots with Tukey whiskers were generated using Prism version 5.02 (Graphpad Software, La Jolla, CA, USA). Medians and ranges were calculated. The distribution of our data was non‐normal. For this reason, comparisons of continuous variables between edentulous and dentate subjects were analyzed using the Mann–Whitney *U*‐test and comparisons of categorical data were analyzed using the chi‐square test. Within groups (edentulous or dentate), comparisons between unstimulated and stimulated samples (*F. nucleatum* or PMA), and oPMNs vs. cPMNs, were performed using the Wilcoxon signed‐rank test. Values of *P *<* *0.05 were considered statistically significant.

## Results

### Characteristics of subjects

The study population consisted of 21 edentulous participants and 20 dentate participants (Table 1) with a median age of 66 yr for the edentulous group and 63 yr for the dentate group (*P *=* *0.043). The majority of participants were male (13 were edentulous and 15 were dentate, *P *=* *0.505). Among the edentulous participants, four were current smokers, while one participant from the dentate group was a current smoker (*P *=* *0.343). Twelve edentulous individuals reported medication use compared with two dentate participants (*P *=* *0.003).

**Table 1:** Summary of participants’ background characteristics, dental features and counts of oral polymorphonuclear neutrophils (oPMNs) and circulatory system polymorphonuclear neutrophils (cPMNs)

Variable	Edentulous subjects (*n* = 21)	Dentate subjects (*n* = 20)	*P*‐value^a^
Age (yr)	66 (44–82)	63 (49–77)	**0.043**
Males	13 (62)	15 (75)	0.505
Smokers	4 (19)	1 (5)	0.343
Medication users	12 (57)^b^	2 (10)^c^	**0.003**
Last tooth extracted			
<5 yr ago	1 (5)	NA	
>5 yr ago	20 (95)	NA	
Last modification denture			
<5 yr ago	11 (52)	NA	
>5 yr ago	10 (48)	NA	
Oral discomfort^d^	2 (10)	NA	
Counts			
oPMNs^e^	0.3 × 10^6^ (0.1–1.0)	0.5 × 10^6^ (0.1–4.5)	**0.030**
cPMNs^f^	13.0 × 10^6^ (3.7–33.4)	9.7 × 10^6^ (0.7–29.4)	**0.022**

Values represent median (range) or *n* (%). NA, not applicable.

a Continuous variables were analyzed using the Mann–Whitney *U*‐test and categorical data were analyzed using the *χ* ^2^ test. Values of *P *<* *0.05 were considered statistically significant and are highlighted in bold.

b Self‐reported medication use: anti‐arrhythmic drug (*n* = 1), antidepressant (*n* = 1), beta blocker (*n* = 1), calcium‐channel blocker (*n* = 1), diuretics (*n* = 3), gastric acid inhibitor/antacid (*n* = 5), cholesterol‐lowering drugs (*n* = 5), calcium supplements (*n* = 1).

c Self‐reported medication use: anti‐hypertensive drugs (*n* = 1), cholesterol‐lowering drugs (*n* = 2).

d Self‐reported denture‐related discomfort; mouth dryness (*n* = 2).

e Total number of oPMNs collected after 4 × 30 s rinsing with 4 min 30 s intermission, after the isolation and purification steps.

f Total cPMNs obtained from a 9‐ml tube of blood, after the isolation and purification steps.

### PMN counts

The results of the oPMN and the cPMN counts are shown in Table 1. After four serial rinses, the median number of oPMNs isolated from edentulous participants (0.3 × 10^6^ oPMNs) was lower than the median number isolated from dentate participants (0.5 × 10^6^ oPMNs) (*P *=* *0.030). The yield of cPMNs from a 9‐ml tube of blood was higher for edentulous participants (median: 13.0 × 10^6^ cPMNs) than for dentate participants (median: 9.7 × 10^6^ cPMNs) (*P *=* *0.022). One blood sample from an edentulous subject was discarded because of technical failure.

### Assessment of membrane integrity

**Figure 1:**
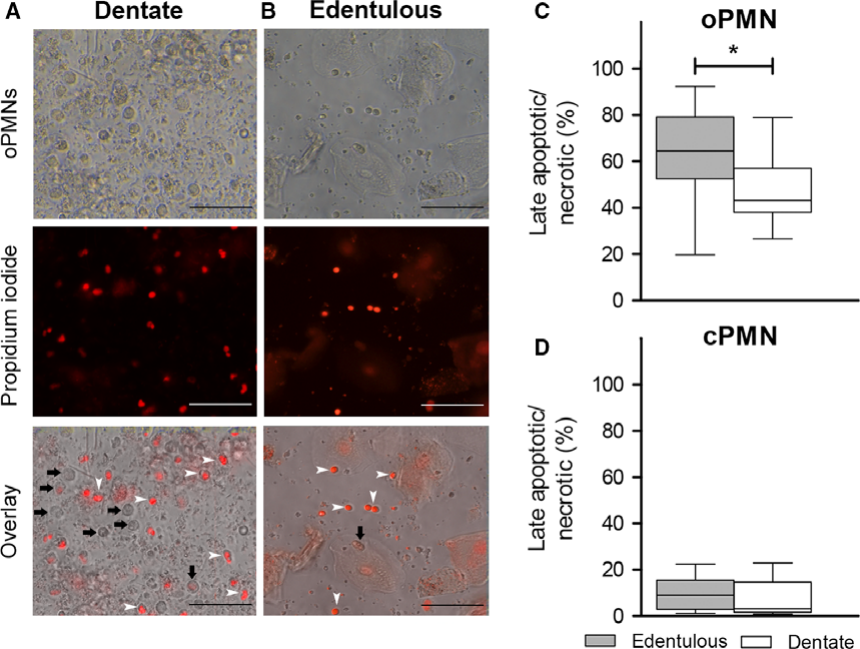
Membrane integrity assessment. Polymorphonuclear neutrophils (PMNs) isolated from the oral cavity of dentate (A) and edentulous (B) participants, and the results of the flow‐cytometric analysis of propidium iodide (PI) uptake for oral PMNs (oPMNs) (C) and circulatory system PMNs (cPMNs) (D). Fewer oPMNs were present in the samples originating from the edentulous participants. Black arrows indicate oPMNs, white arrows represent corresponding propidium iodide‐positive (PI ^+^) cells, indicating late apoptotic/necrotic cells. Boxplots show medians, interquartile ranges, and Tukey whiskers. *P =* *0.002 (Mann–Whitney *U*‐test). Scale bar = 50 *μ*m.

The oPMNs and the results of the flow cytometric membrane integrity assessment are presented in Fig. 1 and Table S1. A higher percentage of late apoptotic/necrotic oPMNs was found for edentulous participants (median: 64.5% oPMNs) than for dentate participants (median: 43.1% oPMNs) (*P = *0.002) (Fig. 1C). In contrast, uptake of PI by cPMNs was low in all samples, and did not differ significantly between edentulous and dentate participants (*P = *0.301) (Fig. 1D). Consequently, the percentage of late apoptotic/necrotic cells was higher among oPMNs than among cPMNs for both the edentulous and the dentate participants (both *P *<* *0.001).

### Cell activation status

**Figure 2:**
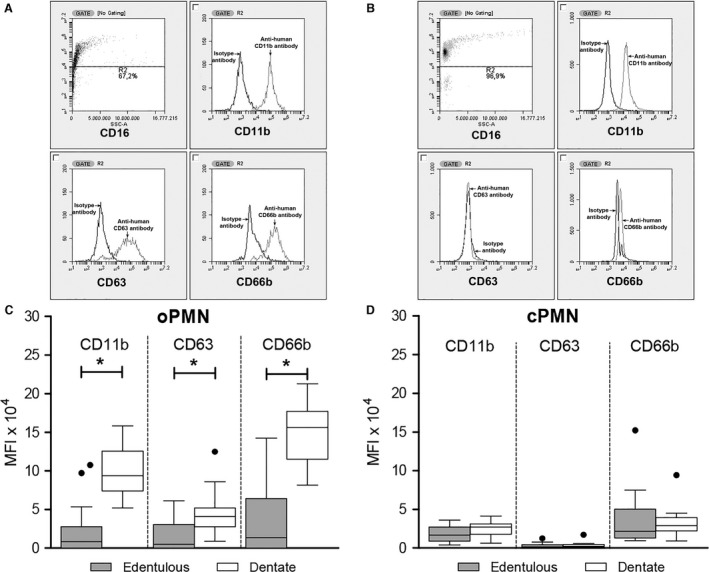
Cell activation status. Immunohistochemistry of oral polymorphonuclear neutrophil (oPMN) and circulatory system polymorphonuclear neutrophil (cPMN) populations. Polymorphonuclear neutrophils (PMNs) were gated based on CD16 expression and sideward scatter. Representative examples show a distinct subset of CD16^+^ cells, indicating mature oPMNs (A) and cPMNs (B). Flow‐cytometric analysis of the cell‐activation markers CD11b, CD63, and CD66b on oPMNs (C) and cPMNs (D) are presented as mean fluorescence intensity (MFI). For each CD marker, corresponding isotype‐control antibodies were used to determine autofluorescent signals, which were subtracted from the MFI. Boxplots show medians, interquartile ranges, and Tukey whiskers, and black dots represent outliers. All *P <* *0.05 (Mann–Whitney *U*‐test).

CD16^+^ cells, indicating mature PMNs, were analyzed for the expression of cell‐activation markers CD11b, CD63, and CD66b using flow cytometry. The results of the immunohistochemistry analyses are presented in Fig. 2 and Table S1. In dentate participants, the median MFI of oPMNs was 9.4 × 10^4^ for CD11b, 4.1 × 10^4^ for CD63, and 15.6 × 10^4^ for CD66b. A different pattern of expression was observed in edentulous participants, with all activation markers being present at very low levels on oPMNs, namely median MFI of 0.8 × 10^4^ for CD11b, 0.5 × 10^4^ for CD63, and 1.4 × 10^4^ for CD66b. Seven of 21 of the edentulous individuals showed no expression of the activation markers on oPMNs (i.e. the binding of the specific mAbs against CD11b, CD63, and CD66b was equal to or lower than the background binding of the isotype‐control antibodies). Consequently, expression of the CD markers was lower on oPMNs from edentulous participants than on oPMNs from dentate participants (all *P *<* *0.001) (Fig. 2A). All three markers were expressed at low levels on cPMNs, with no statistical differences between edentulous and dentate participants (Fig. 2B). In dentate participants, oPMNs were activated to a higher degree compared with cPMNs (CD11b and CD63, both *P *<* *0.001; CD66b, *P *=* *0.001) (Table S1). In contrast, in edentulous participants, the expression of CD11b and CD66b did not differ between oPMNs and cPMNs, whereas CD63 was expressed at a higher level on oPMNs than on cPMNs (*P *=* *0.011) (Table S1).

### ROS production assay

**Figure 3:**
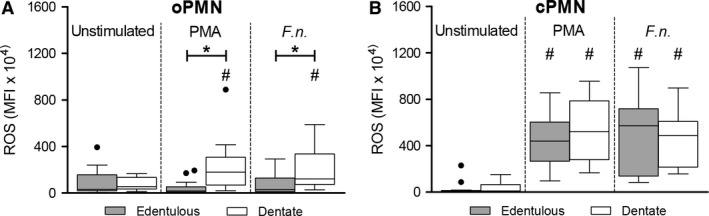
Reactive oxygen species (ROS) production. Results for the flow‐cytometric analysis of ROS production from unstimulated and stimulated oral polymorphonuclear neutrophils (oPMNs) (A) and circulatory system polymorphonuclear neutrophils (cPMNs) (B). Stimulated polymorphonuclear neutrophils (PMNs) were incubated with either phorbol myristate acetate (PMA) or *Fusobacterium nucleatum* (*F.n*.). Boxplots show medians, interquartile ranges, and Tukey whiskers, and black dots represent outliers. *P <* *0.05 (Mann–Whitney *U*‐test), ^#^ *P *<* *0.05 (Wilcoxon signed‐rank test) representing comparison with the corresponding unstimulated samples.

The results for the expression of the ROS production are shown in Fig. 3 and Table S1. The unstimulated oPMNs produced comparable median levels of ROS in the edentulous participants (median MFI: 32.0 × 10^4^) and the dentate participants (median MFI: 54.9 × 10^4^) (Fig. 3A). In the dentate group, the oPMNs were capable of responding to both PMA (median MFI: 179.9 × 10^4^) and *F. nucleatum* (median MFI: 122.1 × 10^4^), which resulted in higher levels of ROS compared with unstimulated oPMNs (PMA and *F. nucleatum* both *P *=* *0.001) (Fig. 3A). In contrast, no upregulation was observed in oPMNs from edentulous participants, in response to either PMA (median MFI: 16.2 × 10^4^) or *F. nucleatum* (median MFI: 28.3 × 10^4^) (Fig. 3A). As a result, the stimulated ROS levels were lower for oPMNs from edentulous participants compared with the levels of ROS produced by the oPMNs from dentate participants (PMA, *P *<* *0.001; *F. nucleatum*,* P *=* *0.002) (Fig. 3A).

The unstimulated cPMNs showed low levels of ROS expression, with no differences between the edentulous participants (median MFI: 7.7 × 10^4^) and the dentate participants (median MFI: 11.4 × 10^4^ (Fig. 3B). Stimulation with PMA and *F. nucleatum* induced robust upregulation compared with unstimulated cPMNs in both groups (PMA and *F. nucleatum*, both *P *<* *0.001) (Fig. 3B and Table S1). The levels of ROS produced by cPMNs in response to stimulation were comparable in the edentulous (PMA median MFI: 4.4 × 10^6^; *F. nucleatum* median MFI: 5.7 × 10^6^) and the dentate participants (PMA median MFI: 5.2 × 10^6^, *P *=* *0.277; *F. nucleatum* median MFI: 4.9 × 10^6^, *P *=* *0.988).

The unstimulated oPMNs expressed higher levels of ROS compared with the cPMNs, both in the edentulous group (*P *=* *0.001) and the dentate group (Table S1, *P *=* *0.007). However, the ROS levels observed upon stimulation were lower in oPMNs than in cPMNs (PMA and *F. nucleatum*, both *P *<* *0.001) (Table S1).

## Discussion

The edentulous participants had fewer oPMNs compared with the dentate participants. The oPMNs from the edentulous participants presented with compromised membranes and no functional respiratory burst capabilities, whereas the dentate participants presented with functional oPMNs. Of importance was that the functional status of the cPMNs from both the edentulous and dentate individuals was comparable. Our findings suggest that oPMNs are functionally impaired in edentulous participants.

One plausible explanation for the low numbers and altered phenotype of the oPMNs from the edentulous group comes from an anatomical perspective. The site of origin of the oPMN cannot be identical in dentate and edentulous participants. The gingival crevice has long been proposed and accepted as the main port of entry for oPMNs into the oral cavity. In dentate participants, substantial numbers of PMNs (±3.0 × 10^4^ PMNs min^−1^) have been reported to migrate constantly from the blood circulation through crevices around the teeth 24, 25, 26, 27, 28. This migration is facilitated by the presence of the lymphatic vessels and the structure of the junctional epithelium, whose number of epithelial layers decreases in the apical part, together with fewer tight junctions observed between the cells 29, 30, 31. In response to oral microorganisms, proinflammatory molecules are generated within the gingival tissues, leading to increased chemotaxis for PMNs 32, 33. In edentulous participants, both the gingival crevices and the junctional epithelia are lost, which affects the access of PMNs to the oral cavity. A second explanation for the low numbers and the altered oPMN phenotype is that in general, denture wearers harbor a less diverse microbiome and the large majority (i.e. >70%) is heavily colonized with *Candida* species 34, 35. Polymorphonuclear neutrophils have been shown to exhibit diminished defensive activity against *Candida* biofilms, including poor recruitment, impaired ROS production and reduced killing 36, 37. In the current study, edentulous participants also presented with fewer numbers of oPMNs and diminished functionality compared with dentate participants.

It can be discussed whether oPMNs can be found at all in the oral cavity of edentulous participants. However, despite the absence of teeth and gingival crevices, PMNs probably have a surveillance function in the edentulous oral cavity similar to that described for the nasal, lung, or vaginal mucosae, or the intestinal lumen 38, 39, 40, 41. A previous study characterized the immune cells present at various oral sites in healthy participants. These authors demonstrated the presence of migrating PMNs in biopsies from buccal mucosa 42. Another study demonstrated the possibility of active transepithelial migration of PMNs in an in vitro model of the multilayered oral epithelium 43. Combined, these observations strongly suggest that oPMNs serve a surveillance function also in the edentulous oral mucosa. On the other hand, the results in the current study show that the oPMNs in edentulous participants have very limited activation potential, as the levels of primary and secondary granules (CD63 and CD66b) were low and no respiratory burst could be further induced. We speculate that the oPMNs emerging at the interface between the epithelial barrier and the mucosal biofilm have fulfilled their patrolling function, are exhausted or necrotic, and then are shed and consequently appear in the sample rinse fluid. Interestingly, in the present study, the numbers of oPMNs observed in edentulous participants were higher than reported previously 15, 16, 17 and may be related to the different sample collection method. In the present study, all participants were asked to rinse vigorously with a saline solution, in order to dislodge the PMNs attached to the mucosa throughout the oral cavity, whereas various reports isolated oPMNs from unstimulated saliva or by drooling 15, 16, 17. Also, in the present study a calibrated cell‐counting device was used instead of visual identification with a light microscope. The cell‐counting device is not prone to human error and is capable of identifying PMNs in low‐abundance cell suspensions 44. Nevertheless, to end the discussion on the existence of oPMN in edentulous participants, we cannot exclude the possibility that non‐visible transient wounds are an additional source of PMNs in the oral cavity, even though the absence of intra‐oral wounds was confirmed in this study by careful clinical examination.

Analysis of cPMNs was included to account for possible differences already occurring in the blood circulation. Importantly, our data showed comparable cPMNs parameters amongst the participants; only the cPMN numbers were higher in the edentulous participants. We cannot exclude a possible effect of the general health status on the cPMN counts 45. As tooth loss has been proposed as a marker for systemic changes associated with a higher risk for developing chronic disease, including changes in lifestyle and socio‐economic factors, infection, and inflammation 46, 47, 48, 49, 50, 51, it is not surprising that smoking and the use of medication were reported more frequently in edentulous participants.

Altogether, edentulous participants display impaired oral PMNs, despite having functional PMNs in the blood circulation. These oPMNs are present in lower numbers and for the most part are non‐functional compared with those from dentate participants. We suggest that this severely hampered aspect of the ‘normal’ innate immunity in the oral cavity of edentulous participants can make them less resistant to pertubations and more prone to dysbiotic conditions, such as oral mucositis, *Candida* infections, and even oral cancer. These findings underline the importance of the presence and maintenance of the natural teeth where the gingival crevices act as a gateway for preserving the innate defense mechanisms within the oral cavity.

## Conflicts of interest

The authors declare that they have no conflict of interest. The Department of Periodontology is supported by a grant from the University of Amsterdam for research into the focal point ‘Oral Infections and Inflammation’.

## Supporting information


**Table S1.** Overview of results of analysis of oPMNs and cPMNs.
