# Supplementary material for: Impaired polymorphonuclear neutrophils in the oral cavity of edentulous individuals
Source: Eur J Oral Sci. 2017 Aug 19;125(5):371–8. doi: 10.1111/eos.12367 (PMC5601278; doi:10.1111/eos.12367)
Supplement: Supplementary file 1 — Table S1. Overview of results of analysis of oPMNs and cPMNs. [file EOS-125-371-s001.pdf]

Supporting Information

Impaired polymorphonuclear neutrophils in the oral cavity of edentulous individuals

RIJKSCHROEFF P, LOOS BG, NICU EA

Department of Periodontology, Academic Centre for Dentistry Amsterdam, the Netherlands

Table S1. Overview of results of analysis of oPMNs and cPMNs.

	Edentulous subjects			Dentate subjects		
	oPMNs	cPMNs	*P*-value*	oPMNs	cPMNs	*P*-value*
Late apoptotic/necrotic	64.5% (19.9-85.4)	9.1% (1.1-22.5)	**<0.001**	43.1% (29.5-81.4)	3.3% (1.2-29.4)	**<0.001**
Cell activation						
CD11b (MFI x 10^4)	0.8 (0.0-10.8)	1.7 (0.4-3.6)	0.516	9.4 (5.2-15.9)	0.6 (4.1-1.8)	**<0.001**
No expression; n (%)	7 (33.3%)[a]	0		0	0	
CD63 (MFI x 10^4)	0.5 (0.0-6.1)	0.2 (0-1.3)	**0.011**	4.1 (1.0-12.5)	0.2 (0.1-1.7)	**<0.001**
No expression; n (%)	6 (28.6%)[b]	2 (10%)[c]		0	0	
CD66b (MFI x 10^4)	1.4 (0.0-71.7)	2.2 (1.0-60.8)	0.452	15.6 (8.2-21.3)	2.9 (0.9-30.6)	**0.001**
No expression; n (%)	6 (28.6%)[d]	0		0	0	
ROS (MFI x 10^4)						
Unstimulated	32.0 (4.3-394.2)	7.7 (1.7-230.2)	**0.001**	54.9 (13.0-166.7)	11.4 (2.2-151.8)	**0.007**
PMA	16.2 (2.8-193.9)	438.8 (97.9-856.9)	**<0.001**	179.9 (18.7-888.4)	521.0 (167.6-957.1)	**<0.001**
F.nucleatum	28.3 (11.1-292.4)	571.3 (83.3-1074.2)	**<0.001**	122.1 (27.1-589.3)	487.7 (156.9-898.3)	**<0.001**

Values represent medians (range) or numbers (percentages). MFI, mean fluorescence intensity; PMA, phorbol myristate acetate; *F. nucleatum*, *Fusobacterium nucleatum*. * Wilcoxon Signed Rank test of comparisons between oPMNs and cPMNs. *P*-values <0.05 were considered statistically significant and are highlighted in bold.

[a] χ^2 test, comparison between edentulous and dentate subjects, *P*=0.009.
[b] χ^2 test, comparison between edentulous and dentate subjects, *P*=0.021.
[c] χ^2 test, comparison between edentulous and dentate subjects, *P*=0.487.
[d] χ^2 test, comparison between edentulous and dentate subjects, *P*=0.021.
